# COVID-19 Pandemic and Psychiatric Symptoms: The Impact on Parkinson's Disease in the Elderly

**DOI:** 10.3389/fpsyt.2020.581144

**Published:** 2020-11-27

**Authors:** Delfina Janiri, Martina Petracca, Lorenzo Moccia, Luca Tricoli, Carla Piano, Francesco Bove, Isabella Imbimbo, Alessio Simonetti, Marco Di Nicola, Gabriele Sani, Paolo Calabresi, Anna Rita Bentivoglio

**Affiliations:** ^1^Department of Psychiatry, Fondazione Policlinico Universitario Agostino Gemelli IRCCS, Rome, Italy; ^2^Department of Psychiatry and Neurology, Sapienza University of Rome, Rome, Italy; ^3^Dipartimento Scienze dell'Invecchiamento, Neurologiche, Ortopediche e della Testa-Collo, Fondazione Policlinico Universitario A. Gemelli IRCCS, Rome, Italy; ^4^Institute of Neurology, Università Cattolica del Sacro Cuore, Rome, Italy; ^5^IRCCS Fondazione Don Carlo Gnocchi, Milan, Italy

**Keywords:** COVID-19, Parkinson's disease, depression, psychosis, irritability, delusions, psychiatric symptom, mood stabilizers

## Abstract

**Background:** The coronavirus disease 2019 (COVID-19) pandemic represents a condition of increased vulnerability and frailty for elderly patients with Parkinson's disease (PD). Social isolation may worsen the burden of the disease and specifically exacerbate psychiatric symptoms, often comorbid with PD. This study aimed at identifying risk/protective factors associated with subjective worsening of psychiatric symptomatology during the COVID-19 outbreak in a sample of individuals with PD aged 65 years or older.

**Methods:** Patients with PD routinely followed at the outpatient clinic of Gemelli University Hospital, Rome, were assessed for subjective worsening of psychiatric symptoms through a dedicated telephone survey, after Italy COVID-19 lockdown. Patients' medical records were reviewed to collect sociodemographic and clinical data, including lifetime psychiatric symptoms and pharmacological treatment.

**Results:** Overall, 134 individuals were assessed and 101 (75.4%) reported lifetime psychiatric symptoms. Among those, 23 (22.8%) presented with subjective worsening of psychiatric symptomatology during the COVID-19 outbreak. In this group, the most frequent symptom was depression (82.6%), followed by insomnia (52.2%). Subjective worsening of neurological symptoms (Wald = 24.03, df = 1, *p* = 0.001) and lifetime irritability (Wald = 6.35, df = 1, *p* = 0.020), together with younger age (Wald = 5.06, df = 1, *p* = 0.038) and female sex (Wald = 9.07 df = 1, *p* = 0.007), resulted as specific risk factors for ingravescence of psychiatric presentation. Lifetime pre-existing delusions, having received antipsychotics, and not having received mood stabilizer were also associated with subjective worsening of psychiatric symptomatology due to the COVID-19 pandemic.

**Conclusions:** Individuals with PD and lifetime history of psychiatric symptoms may be exposed to increased vulnerability to the stressful effect of COVID-19 outbreak. Interventions aimed at reducing irritability and mood instability might have an indirect effect on the health of patients with PD during the COVID-19 pandemic.

## Introduction

In a very short time, our world has dramatically changed. The coronavirus disease 2019 (COVID-19) has disrupted normality across the globe, throwing all aspects of life into uncertainty. The situation is even more critical for patients affected by chronic neurological disorders, such as Parkinson's disease (PD). The more widespread challenge is the limited access to adequate care, as a consequence of self-isolation and social distancing that have been enforced on a global level. Elderly patients are particularly exposed to these conditions of increased vulnerability and frailty (1, 2). Based on data from large cohort studies (3) and systematic reviews of the literature (4), there is, to date, no evidence whether elderly individuals with PD are at increased risk for COVID-19, compared to individuals of similar age and with comparable comorbidities. Nevertheless, social isolation, especially if protracted, may worsen the burden of neurological disorders (5). According to this, our group recently demonstrated specific correlations between subjective worsening of neurological symptoms and consequences of social restrictions in a large sample of 2,167 outpatients with chronic neurologic diseases (6).

Psychiatric symptoms are common and disabling conditions in the clinical course of PD (7–9), and they are specifically highly prevalent in elderly patients with PD (10, 11). They include affective disorders, apathy and anhedonia, disorders of sleep and wakefulness, psychosis, and impulse control disorders (7, 12). Psychiatric features are typically multimorbid, characterized by great intra- and inter-individual variability in clinical presentation (8) and may be largely influenced by life stress events (13). The COVID-19 pandemic represents an important stressor associated with the exacerbation of psychiatric symptoms (14, 15). Recent data highlighted higher levels of psychological distress among the general population (16) and increased risk for recurrences and worsening in patients with neuropsychiatric disorders during the COVID-19 outbreak. Accordingly, preliminary reports over the last few weeks demonstrated higher levels of stress, depression, and anxiety in patients with PD compared to healthy controls (HC) (17, 18). They also confirmed impaired quality of life during social restriction (17).

However, studies in elderly people with PD specifically focused on the worsening of psychiatric symptoms due to COVID-19 pandemic are still lacking. Furthermore, insufficient data are available on lifetime risk/protective factors potentially associated with clinical exacerbation. This study aimed at filling these gaps by describing the prevalence of subjective worsening of psychiatric symptomatology during the COVID-19 outbreak, while identifying associated risk/protective factors, in a sample of individuals with PD aged 65 years or older.

## Methods

### Patient Population

We assessed 134 individuals with PD who were regularly followed at the outpatient clinic of the Department of Neurology at Agostino Gemelli University Hospital Foundation IRCCS-Catholic University of the Sacred Heart in Rome. Patients were consecutively enrolled in the study if they had a scheduled visit during the lockdown. Individuals aged 65 years or older, Caucasian, and under stable psychopharmacological treatment for at least 6 months were included. Patients were excluded if they or their legal support administrators were unable to provide informed and valid consent at the time of the assessment. Patients not fluent in Italian, with severe and unstable medical conditions (i.e., not non-stabilized diabetes, oncologic disorders, clinically significant and unstable active gastrointestinal, renal, hepatic, endocrine, or cardiovascular disorder), dementia, or cognitive deterioration according to DSM-5 criteria, and Mini-Mental State Examination (MMSE) score <25 were also excluded from the study.

### Data Collection

Sociodemographic and clinical data before the COVID-19 outbreak (i.e., age at onset of motor symptoms, neurological characteristics, lifetime psychiatric symptoms, and pharmacological treatment) were extracted from patients' medical records. Lifetime psychiatric symptoms were assessed through a semi-structured interview described below. Neurological characteristics were evaluated through the motor examination section of the Unified Parkinson's Disease Rating Scale (UPDRS) disease severity (UPDRS-III) (19) and the disease stage according to Hoehn and Yahr (H & Y) stage (20).

Information related to the impact of COVID-19 on psychiatric symptoms was collected through a telephone survey. The survey started on April 1, 2020, and ended on April 15, 2020. A semi-structured interview was adopted to evaluate the impact of social restrictions on psychiatric burden. The semi-structured interview, carried out by a senior psychologist, was based on current evidence on psychiatric disorders in PD (7), on DSM-5 criteria, and on clinical evaluation (not on simple yes/no answers to structured questions). The wording of the questions could be changed to improve/check understanding, and the final evaluation was also based on information from the caregiver (if available) and from any medical documentation. Specifically, the survey assessed the presence (classified as “yes” or “no”) of depression, apathy/anhedonia, sleep disturbances (insomnia), rapid eye movement (REM) sleep behavior disorders (RBD), irritability, impulse control disorders (ICDs), delusions, and hallucinations. Furthermore, participants were also asked to report subjective worsening of psychiatric symptoms and neurological symptoms. If they reported a worsening of their clinical presentation, they were referred to a multidisciplinary team, composed by neurologists and psychiatrists experienced in the field of movement disorders, to adjust pharmacological treatment. All data collected about past and current psychiatric symptoms were entered in preprinted medical records.

The Survey was reviewed and approved by the Ethics Committee of the Agostino Gemelli University Hospital Foundation IRCCS-Catholic University of the Sacred Heart Ethics Committee, Rome. Because of the biological risks related to the pandemic, participants could not timely provide written informed consent. Therefore, during the phone call, verbal consent for study participation and use of anonymized data was obtained (immediate consent) according to information filed with the Ethics Committee. Participants were informed that written consent would be obtained at the first visit in the hospital (deferred consent).

### Statistical Methods

For the aim of the study, in the analyses, we considered patients with lifetime history of psychiatric symptoms. We compared individuals who reported subjective worsening of psychiatric symptomatology during the COVID-19 outbreak with those reporting symptom stability on demographic characteristics, neurological characteristics (age at onset of motor symptoms, UPDRS-III score, H & Y stage, and worsening of neurological symptoms during the COVID-19 outbreak), lifetime type of psychiatric symptoms, and pharmacological treatment. Analyses used standard univariate/bivariate comparisons of continuous measures (ANOVA) and categorical measures (contingency table/χ^2^) to assess significant differences between groups. The level of significance for the analyses was set at *p* < 0.05. In addition, we used a multivariate logistic regression model to identify lifetime risk and protective factors that significantly differentiated patients who reported (or did not report) worsened psychiatric presentation (considered as the dependent variable). All factors that resulted significant in the univariate analyses were included in the model and considered as independent variables. We examined possible multicollinearity between factors of interest using the variance inflation factor (VIF) indicator obtained from a linear regression analysis.

All the statistical analyses were carried out using the “Statistical Package for Social Science (SPSS)” program, version 25.0 (IBM Co., Armonk, NY).

## Results

**Figure 1:**
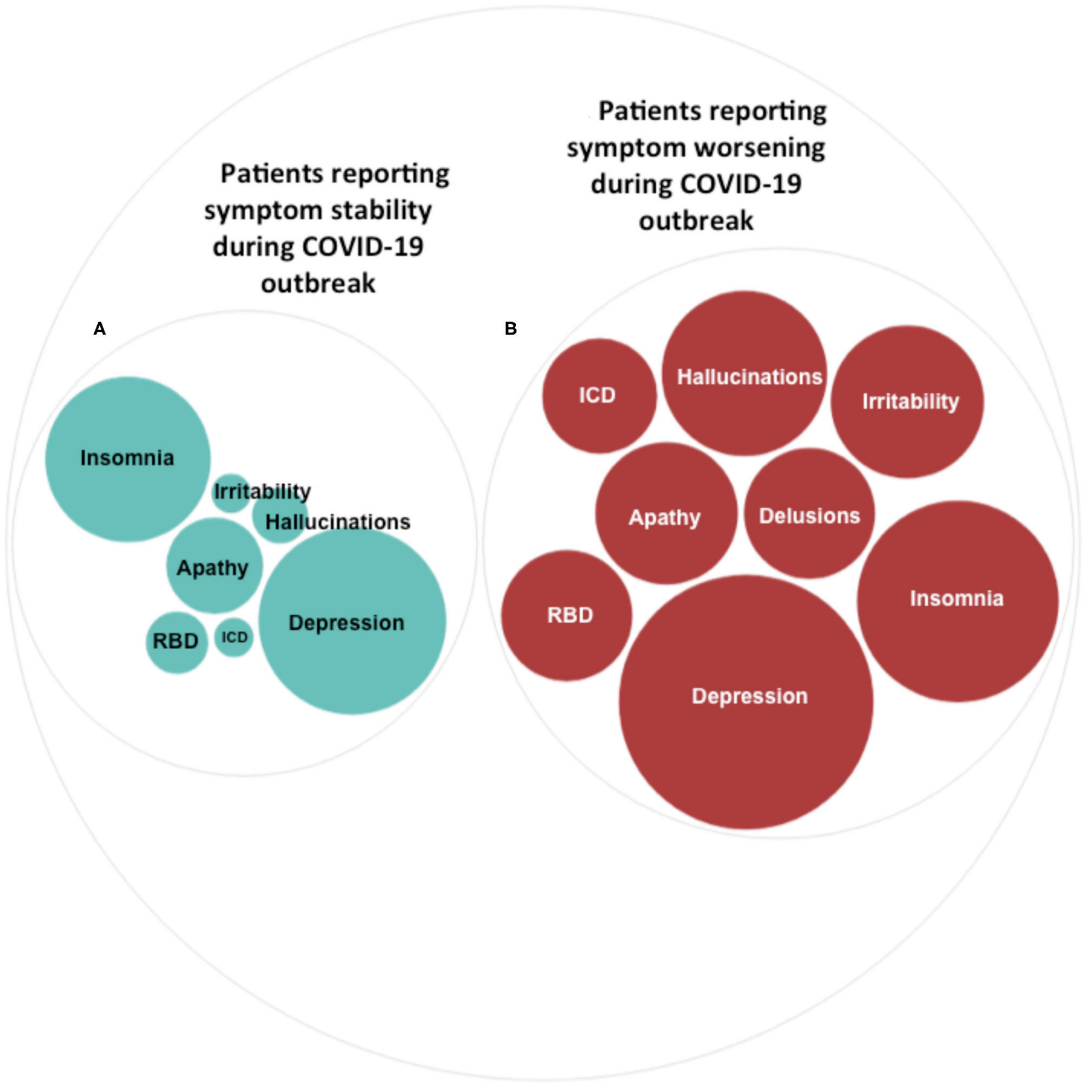
Psychiatric symptoms during the COVID-19 outbreak. Prevalence of different types of psychiatric symptoms in patients reporting symptom stability **(A)** and in patients reporting symptom worsening **(B)** during the COVID-19 outbreak. The size of the circles indicates the percentages (%) of individuals in each group reporting specific psychiatric symptoms. Percentages are detailed in Table 1.

In the total sample (*n* = 134), 101 (75.4%) patients reported lifetime psychiatric symptoms. Among those, 23 (22.8%) reported subjective worsening of psychiatric symptomatology during the COVID-19 outbreak. The most frequent symptom among patients reporting worsened symptoms was depression (82.6%), followed by insomnia (52.2%) (Table 1 and Figure 1). In terms of demographic features, the two groups differed in sex (χ^2^ = 8.87, df = 1, *p* = 0.003) and age (*F* = 9.38, df = 1, *p* = 0.003) (Table 2). Specifically, most participants reporting symptom exacerbation were women (*n* = 16, 69.6%) and younger (mean age = 69.78 years, sd = 4.38) than those reporting symptom stability (female: *n* = 27, 34.6%; mean age = 73.94 years, sd = 6.08) (Table 2).

**Table 1:** Current psychiatric symptomatology in patients reporting symptoms worsening or stability during the COVID-19 outbreak.

**Current psychiatric symptoms**	**Patients reporting symptoms worsening during the COVID-19 outbreak (*n* = 23) *N* (%)**	**Patients reporting symptoms stability during the COVID-19 outbreak (*n* = 78) *N* (%)**
Depression	19 (82.6)	35 (44.9)
Apathy/anhedonia	6 (26.1)	9 (11.5)
Insomnia	12 (52.2)	27 (34.6)
RBD	5 (21.7)	4 (5.1)
Irritability	7 (30.4)	1 (1.3)
ICD	4 (17.4)	1 (1.3)
Delusions	5 (21.7)	0 (0)
Hallucinations	8 (34.8)	3 (3.8)

*RBD, rapid eye movement (REM) sleep behavior disorders; ICD, impulse control disorders*.

**Table 2:** Lifetime features associated with symptoms worsening or stability during the COVID-19 outbreak.

	**Patients reporting symptoms worsening during the COVID-19 outbreak (*n* = 23) *N* (%)**	**Patients reporting symptoms stabilityduring the COVID-19 outbreak (*n* = 78) N (%)**	**χ^2^**	**df**	***p***
Age: mean ± (SD)	69.78 (4.38)	73.94 (6.08)	9.38	1	0.003^*^
Sex: *n* (%)	16 (69.6)	27 (34.6)	8.87	1	0.003^*^
**Neurological characteristics**					
Age at onset of motor symptoms: mean ± (SD)	58.56 (8.62)	62.75 (9.84)	3.37	1	0.069
UPDRS-III: mean ± (SD)	30.82 (13.22)	26.54 (11.83)	2.19	1	0.14
H & Y stage: mean ± (SD)	3.13 (0.99)	2.81 (0.94)	1.96	1	0.16
Subjective worsening of neurological symptoms during the COVID-19 outbreak: *n* (%)	18 (84.6)	12 (21.7)	33.60	1	<0.001^*^
**Lifetime type of psychiatric symptoms**					
Depression: *n* (%)	17 (73.9)	44 (56.4)	2.27	1	0.13
Apathy/anhedonia: *n* (%)	6 (26.1)	29 (37.2)	0.96	1	0.32
Insomnia: *n* (%)	13 (56.5)	54 (69.2)	1.28	1	0.25
RBD: *n* (%)	9 (39.1)	28 (35.9)	0.08	1	0.77
Irritability: *n* (%)	12 (52.2)	13 (16.7)	12.02	1	0.001^*^
ICD: *n* (%)	6 (26.1)	14 (17.9)	0.74	1	0.38
Delusions: *n* (%)	4 (17.4)	3 (3.8)	5.05	1	0.02^*^
Hallucinations: *n* (%)	3 (13.0)	15 (19.2)	0.46	1	0.49
**Pharmacological treatment**					
L-DOPA: *n* (%)	23 (100)	73 (93)	1.55	1	0.21
IMAO: *n* (%)	16 (69.6)	49 (62.8)	0.35	1	0.55
ICOMT: *n* (%)	7 (30.4)	13 (16.7)	2.12	1	0.14
Dopamine agonists: *n* (%)	11 (47.8)	33 (42.3)	0.22	1	0.63
Antidepressants: *n* (%)	8 (34.8)	27 (34.6)	0	1	0.98
Mood stabilizers: *n* (%)	1 (4.3%)	20 (25.6)	4.89	1	0.02^*^
Antipsychotics: *n* (%)	11 (47.8)	17 (21.8)	6.00	1	0.01^*^

*χ^2^, Chi-square tests; df, Degrees of freedom*;

* *significant p; UPDRS-III, the Unified Parkinson's Disease Rating Scale disease severity part III; H & Y stage, Hoehn and Yahr stage; RBD, rapid eye movement (REM) sleep behavior disorders; ICD, impulse control disorders; L-DOPA, levodopa; IMAO, monoamine oxidase inhibitors; ICOMT, catechol-O-methyltransferase-inhibitors*.

Subjective worsening of neurological symptoms along with lifetime pre-existing irritability and delusions, having received antipsychotics, and not having received mood stabilizer were associated with subjective worsening of psychiatric symptomatology during the COVID-19 outbreak (Table 2). Multivariate logistic regression specified that reporting neurological symptoms worsening during the pandemic (Wald = 24.03, df = 1, *p* = 0.001) and lifetime irritability (Wald = 6.35, df = 1, *p* = 0.020), together with younger age (Wald = 5.06, df = 1, *p* = 0.038) and female sex (Wald = 9.07 df = 1, *p* = 0.007), were associated with an increasing likelihood of exhibiting worsening of psychiatric symptoms during the COVID-19 pandemic. The logistic regression model was statistically significant (χ^2^ = 65.8, *p* < 0.001) and correctly classified 73.0% of cases. There was no significance of multicollinearity, as indicated by the fact that VIF of all variables of interest was <2.

## Discussion

The COVID-19 pandemic has forced national health systems to rapidly set priorities in medical care, and this led to dramatic consequences for many patients with chronic conditions, including those with PD (3). The increased vulnerability of the elderly and those with comorbidities, along with the increased prevalence of PD with age, raises concerns about the potentially negative impact of the COVID-19 outbreak on people living with movement disorders (5). Psychiatric symptoms, in particular, could be greatly influenced by the social isolation imposed by the COVID-19 pandemic (14).

In our sample, up to 22.8% of patients with PD experienced worsening of their psychiatric clinical condition during the COVID-19 outbreak. The COVID-19 pandemic is profoundly modifying individuals' routines. Such drastic changes require a flexible adaptation to novel circumstances, a cognitive process partly related to/dependent on normal dopaminergic functioning (21, 22). A growing body of evidence suggests that many patients with PD may experience both cognitive and motor inflexibility, as a result of nigrostriatal dopamine depletion that is involved in the pathophysiological substrate of the disorder (23, 24). This might have a two-sided explanation. On the one hand, it has been hypothesized that dopamine-dependent adaptation sub-serves flexible coping mechanisms to environmental stressors (23, 25). On the other hand, increased psychological stress can temporarily worsen various motor symptoms, including tremor, freezing of gait, or dyskinesias, and reduce the efficacy of dopaminergic medication (26, 27).

Consistently with available evidence (28), a relevant percentage of individuals with PD experienced subjective worsening of neurological symptomatology as a result of the COVID-19 outbreak. Besides, worsening of motor symptoms was the most sensitive clinical risk factor for ingravescence of psychiatric symptoms in patients with PD during the COVID-19 pandemic. Different reasons explaining the negative effect of lockdown on PD motor symptoms have been suggested, including increasing levels of stress that could worsen motor symptoms as well as the discontinuation of physiotherapy and/or reduction in physical activity (6). The relationship between psychiatric symptoms and PD follows a vicious cycle, with the presence of psychiatric disorder increasing the risk of PD, and vice versa. For instance, there is evidence that patients with PD who experience on–off motor fluctuations are also more likely to encounter fluctuations in mood and energy levels. Mood swings are not entirely linked to dopaminergic dosing or PD neurodegeneration but are also observed in patients with a pre-existing psychiatric history or concurrent use of psychiatric medication, suggesting these may in fact be part of a larger symptom constellation (29).

The most frequent symptom in the group of patients presenting with worsening of psychiatric conditions was depression, which has been reported by up to 82.6% of individuals (Table 1 and Figure 1). Depression has been shown to be the most common psychiatric symptom in patients with PD and has been indicated as a specific risk factor for developing the disease (30). One of the largest sample studies to date, using data from a matched cohort of 23,180 participants (4,634 patients with depression and 18,544 control patients), reported that patients with depression were 3.24 times more likely to develop PD compared with the control patients (31). Depression in PD is likely to result from a complex interaction of environmental and neurobiological factors. Neuroimaging analyses suggest that patients with PD reporting depression specifically exhibited widespread disruptions in both function and structure (32). Abnormalities have been primarily reported in subcortical nuclei and prefrontal–temporal–limbic circuits (33–35). Interestingly, the same brain networks have been highlighted as specific targets of stress-induced mood symptomatology (36–38).

Our results also found pre-existing lifetime irritability as a significant clinical risk factor for psychiatric symptom worsening in patients with PD during the COVID-19 pandemic. This is in line with previous observations specifically linking mood instability with irritability (39). Irritability, although often ignored by clinicians, is part of a strong principal factor of major depression (40), and it is associated with greater outcome severity and lower quality of life (40). Lifetime presence of delusions was also associated with symptom worsening during the COVID-19 outbreak. These results could indirectly suggest a more severe and susceptible phenotype in this group of patients. Psychosis in PD is associated with reduced quality of life and worse prognosis and is an independent predictor of increased mortality (41). According to this, although the biological etiology of psychosis in PD has not yet been clearly understood, previous studies hypothesized that psychotic symptoms formation might be linked with hypersensitivity of mesocorticolimbic dopaminergic receptors, cholinergic denervation, serotonergic/dopaminergic imbalance, and neurodegeneration of widespread limbic, paralimbic, and neocortical gray matter (42, 43).

In our study, individuals who developed psychiatric clinical deterioration were significantly more likely to be women and younger as compared to patients who did not present worsening of psychiatric symptoms. Gender is an important biological determinant of vulnerability to psychosocial stress, in addition to genetic, socio-cultural, hormonal, and developmental factors (16). Our results indicate that males are, to a certain degree, less likely to develop psychological symptoms in the face of a stressful event. This is in line with a recent review on mental health consequences of the COVID-19 pandemic, which reports higher risk of psychiatric symptoms and/or low psychological well-being in females compared to males (44). The same study indicated that findings on age as a risk factor for COVID-19-related psychological distress were inconsistent (44). In our study, we found that older age (in the age group of >65 years old) might be a protective factor against psychiatric clinical exacerbation in PD during the COVID-19 pandemic. We may speculate that this could be associated with the observed reduced behavioral reservoir of old individuals, which include psychiatric behaviors (45).

Psychiatric symptom worsening was also associated with a higher lifetime use of antipsychotics and a lower lifetime use of mood stabilizers. The higher rate of antipsychotics indicate worse disease clinical course, particularly with regard to delusions and hallucinations, and suggest higher instability of psychiatric symptoms in this group of patients (46). On the other hand, the higher rate of mood stabilizers may be linked to a potential protective effect of mood stabilization. In our sample, in particular, the use of mood stabilizers could have mitigated mood instability related to irritability through specific biological mechanisms. According to this, there is evidence that the benefits of mood stabilizers extend beyond affective stabilization (47–49) and include neuroprotection against several neuropsychiatric condition (48, 50, 51). Besides, mood stabilizers may prevent individuals with PD from experiencing abrupt shifts in mood, energy, behavior, and thinking when facing stressful events, which, in turn, may promote resilience (52).

Before presenting our conclusions, we must acknowledge some issues that might limit the generalizability of our results. First, the study has a cross-sectional design and lacks longitudinal follow-up of patients reporting symptoms worsening during the pandemic after the adjustment of their pharmacological treatment. Furthermore, the mental health impact of the COVID-19 outbreak on patients with PD could change during time. Therefore, long-term psychological implications of this population warrant further investigation. Second, the lack of standardized questionnaires for psychiatric symptoms is another limitation of our study. However, all patients underwent a detailed anamnesis, which included the evaluation of psychiatric clinical aspects. Third, the survey design required telephone contact rather than face-to-face assessment; as a consequence, the interview may be influenced by uncontrolled and recall bias.

In conclusion, our study highlighted that patients with PD are at increased risk of experiencing the negative sequelae of the pandemic in terms of both increased stress and limited access to standard neurological care, which can, in turn, adversely affect their psychiatric features. Depression in particular resulted as the most prevalent psychiatric symptom reported by patients presenting with clinical worsening. Our findings suggest that interventions aimed at reducing irritability and mood instability, such as the use of mood stabilizers, might have an indirect effect on the health and well-being of patients with PD during the COVID-19 pandemic.

## Clinical Vignette

We describe the case history of a 56 year-old patient, male, affected by PD, who developed an impressive psychosis during the lockdown period. Disease onset was in 2008 at the age of 44 with akinesia on his left leg and clumsiness in the same side. Except for motor symptoms of disease, he also showed a prodromal hyposmia. No other relevant diseases were reported. A DAT scan confirmed diagnosis in 2009. He started with rasagiline 1 mg qd and ropinirole, tapered up to 20 mg qd during the following years, with a marked improvement of his motor symptoms. Further on, trihexyphenidyl and amantadine were added to his therapy. Since 2011, he has showed mild impulsive compulsive behaviors, i.e., increased libido and compulsive hobbism. In 2017, he reported his first psychotic episode, characterized by persecutory delusions and auditory hallucinations. Ropinirole was tapered off and substituted by levodopa, while antipsychotic therapy with quetiapine up to 125 mg was started, with a gradual improvement of his psychiatric symptoms. In 2019, he stopped quetiapine and started psychotherapy and a physiotherapy program. He also reported motor fluctuations with mild wearing-off of levodopa therapy.

During 2019, the patient asked to change work position. Then, on February 2020, he started working with a new team and initially reported concentration and learning difficulties. On March 9, at the beginning of the Italian lockdown, the patient found himself at home alone in social isolation, without contacts with his working team. On May 2020, he started to show the first signs of a severe psychosis, characterized by psychomotor agitation, auditory hallucinations, and persecutory delusions. The persecutor was identified as a colleague who had had a past love story with his wife. Along with those symptoms, the patient reported instability of mood with irritability and insomnia. At psychiatric assessment, the patient scored 37 on the Young Mania Rating Scale (YMRS) (53). The patient showed no insight into his condition. At neurological examination, he showed mild hypomimia and hypophonia, slight and occasional rest tremor on his left hand, slight rigidity affecting the left extremities, slowing gait with reduced left arm swing, global bradykinesia with reduced amplitude, and slightly slow hand movements. The motor examination section of the Unified Parkinson's Disease Rating Scale (UPDRS) had a total score of 14. On May 25, rasagiline was stopped, and levodopa and amantadine were reduced. Simultaneously, he was started again with quetiapine 25 mg at bedtime, without amelioration. Then, a multidisciplinary team, composed of neurologists and psychiatrists experienced in the field of movement disorders, started to manage the patient's care with a daily clinical follow-up. On June 16, the clinical team stopped quetiapine and prescribed clozapine 12.5 mg at bedtime, and then increased to 25 mg, which induced after only 3 days a marked and impressive improvement of psychotic symptoms (YMRS = 9), which was stable over the next month.

## Data Availability Statement

The raw data supporting the conclusions of this article will be made available by the authors, without undue reservation.

## Ethics Statement

The studies involving human participants were reviewed and approved by Ethics Committee of the Agostino Gemelli University Hospital Foundation IRCCS-Catholic University of the Sacred Heart Ethics Committee, Rome. Written informed consent for participation was not required for this study in accordance with the national legislation and the institutional requirements.

## Author Contributions

DJ, MP, PC, and AB designed the study and wrote the protocol. DJ, MP, LM, CP, FB, II, AS, MD, GS, PC, and AB managed the literature searches and analyses. DJ, MP, and LM performed the statistical analysis and wrote the first draft of the manuscript. All authors contributed to and have approved the final manuscript.

## Conflict of Interest

The authors declare that the research was conducted in the absence of any commercial or financial relationships that could be construed as a potential conflict of interest. The handling editor declared a shared affiliation with one of the authors DJ at time of review.
